# Comparison of the glidescope®, flexible fibreoptic intubating bronchoscope, iPhone modified bronchoscope, and the Macintosh laryngoscope in normal and difficult airways: a manikin study

**DOI:** 10.1186/1471-2253-14-10

**Published:** 2014-02-28

**Authors:** Adrian Langley, Gabriel Mar Fan

**Affiliations:** 1 Department of Anaesthesia and Pain Management, QE II Jubilee Hospital, Metro South HHN, Coopers Plain, QLD 4108, Australia

## Abstract

**Background:**

Smart phone technology is becoming increasingly integrated into medical care.

Our study compared an iPhone modified flexible fibreoptic bronchoscope as an intubation aid and clinical teaching tool with an unmodified bronchoscope, Glidescope® and Macintosh laryngoscope in a simulated normal and difficult airway scenario.

**Methods:**

Sixty three anaesthesia providers, 21 consultant anaesthetists, 21 registrars and 21 anaesthetic nurses attempted to intubate a MegaCode Kelly™ manikin, comparing a normal and difficult airway scenario for each device. Primary endpoints were time to view the vocal cords (TVC), time to successful intubation (TSI) and number of failed intubations with each device. Secondary outcomes included participant rated device usability and preference for each scenario. Advantages and disadvantages of the iPhone modified bronchoscope were also discussed.

**Results:**

There was no significant difference in TVC with the iPhone modified bronchoscope compared with the Macintosh blade (P = 1.0) or unmodified bronchoscope (P = 0.155). TVC was significantly shorter with the Glidescope compared with the Macintosh blade (P < 0.001), iPhone (P < 0.001) and unmodified bronchoscope (P = 0.011). The iPhone bronchoscope TSI was significantly longer than all other devices (P < 0.001). There was no difference between anaesthetic consultant or registrar TVC (P = 1.0) or TSI (P = 0.252), with both being less than the nurses (P < 0.001). Consultant anaesthetists and nurses had a higher intubation failure rate with the iPhone modified bronchoscope compared with the registrars. Although more difficult to use, similar proportions of consultants (14/21), registrars (15/21) and nurses (15/21) indicated that they would be prepared to use the iPhone modified bronchoscope in their clinical practice. The Glidescope was rated easiest to use (P < 0.001) and was the preferred device by all participants for the difficult airway scenario.

**Conclusions:**

The iPhone modified bronchoscope, in its current configuration, was found to be more difficult to use compared with the Glidescope® and unmodified bronchoscope; however it offered several advantages for teaching fibreoptic intubation technique when video-assisted bronchoscopy was unavailable.

## Background

Fibreoptic intubation has long been considered the gold standard intubation technique in patients with an anticipated or known difficulty airway or as a rescue device in failure to intubate but able to ventilate scenarios [1]. Fibreoptic intubation can be a difficult skill to teach, learn and maintain [2]. Since the late 1990’s advances in video technology and fibreoptics has resulted in an increasing number of commercially available video laryngoscopes. Several studies have demonstrated that video laryngoscopes generally provide a better view of the glottis and have higher success rates of intubation compared with the traditional Macintosh blade in patients with a predicted difficult airway. Video laryngoscopes have the additional advantage of less movement of the cervical spine, and are potentially less traumatic; however, these devices may fail secondary to trismus and oropharyngeal tumors, infection or foreign bodies resulting in difficulty inserting the blade. Active bleeding may obscure the view. The presence of airway pathology from previous surgery, a local mass, or radiation treatment are the strongest predictors of Glidescope® failure [3].

Technology is becoming increasingly integrated into medical care. Smart phones, defined as ‘a mobile phone that is able to perform many of the functions of computer devices’, have developed rapidly over the last decade becoming smaller, faster, with improved storage capacity, optical resolution and camera functionality [4]. Reported applications for smart phones as biomedical monitors include interfacing them with oximeters, stethoscopes and microscopes. In anaesthetic practice smart phones have been used for measurement of tilt in obstetric anaesthesia, case log book, aid to resuscitation, education, distraction therapy for children undergoing gas induction, billing, pharmacokinetic modelling and assessment of neuromuscular function [5-7]. Although some of the currently available video laryngoscopes and mobile fibreoptic bronchoscopes can record images and video they are typically more expensive than a smart phone, are larger, may be less available, and lack the telecommunication and data capabilities unique to a mobile phone.

The aim of this study was to assess the usefulness of the iPhone as an adjunct aid to assist in fibreoptic intubation and clinical teaching in a difficult airway scenario when a screen for video-assisted bronchoscopy was unavailable. We recruited non medical anaesthetic personnel to account for variables that may influence performance; including fibreoptic technique, level of experience and familiarity with the iPhone.

## Methods

**Figure 1:**
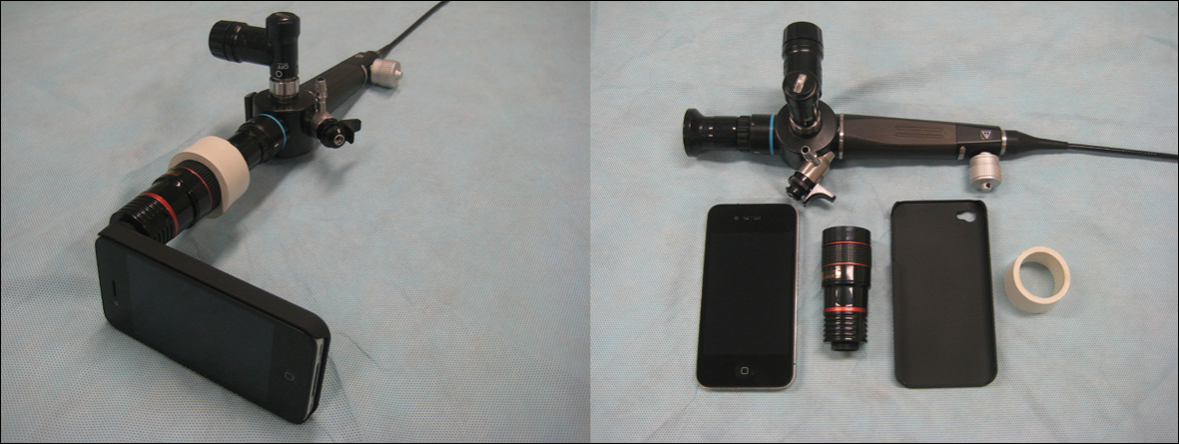
Fibreoptic bronchoscope with iPhone, lens, and rubber attachment.

After obtaining local Ethics Committee (Metro South Health Service District) approval and written informed consent, a total of 63 participants were enrolled into this study. These included 21 consultant anaesthetists, 21 anaesthetic registrars and 21 anaesthetic nurses. All participants had at least 6 months clinical experience in anaesthesia. Participation was voluntary and all data anonymized. Data was collected from each participant including; gender, age, previous anaesthetic experience (years) and level of training, number of estimated intubations with the Macintosh blade, Glidescope®, flexible fibreoptic bronchoscope and their familiarity with smart phones.The study design was a randomized crossover design. The Glidescope® (adult large blade), flexible fibreoptic intubating scope size 3.8 mm (Olympus America Inc, Center Valley, PA, USA) and iPhone (Cupertino, California, USA) modified bronchoscope (iPMFB) were compared to the Macintosh metal blade size 4. The iPhone modification consisted of an iPhone 4.0 attached to an Eye Scope mobile zoom lens. The device was connected to the eye piece of the flexible fiberoptic bronchoscope by a white rubber door stop with the end removed (Figure 1). The endotracheal tube (ETT) was secured to the bronchoscope with removable tape prior to commencement of timing and was preloaded with a rigid stylet formed into the shape of a hockey stick for use with the Glidescope® (Glidescope® Rigid Stylet, BC, Canada).

Before starting the study each participant was given a standardized demonstration of the intubation devices by one of the investigators. This included an explanation and oral instructions on how to use them. The participants were then shown the vocal cords with each device and the recommended technique to successfully intubate the MegaCode Kelly™ manikin (Laerdal Medical AS, Stavanger, Norway). The participants were allowed to practice with each device until one successful intubation had been achieved in the normal airway setting. All intubations were performed with a size 7.0 mm cuffed Portex (Smiths Medical, Kent, UK) ETT and the cuff was inflated and deflated with a 20 ml BD syringe (BD Drogheda, Ireland). The ETT was lubricated with Laerdal Airway Lubricant for training manikins before each intubation attempt. No time limit was placed on the practice intubation attempt. Study volunteers participated with only one of the investigators present. All intubations were performed orally.

Participants performed tracheal intubation with each device in a normal and simulated difficulty airway. The difficult airway was simulated with ‘neck immobilization’ using a hard collar. The Macintosh blade was always the first device used in each scenario. The sequence in which each participant used the other devices was randomized using a Latin square. Each participant used the devices in the same sequence in both scenarios.

The primary endpoints were the time to view the vocal cords (TVC), time taken for successful intubation (TSI) and the number of attempts at intubation. A failed intubation attempt was defined as failure to place the ETT between the cords, more than 3 attempts at intubation, or an attempt requiring > 180 seconds to perform. Timing commenced when the intubation device entered the ‘mouth’ of the manikin. An interim time was taken when the participant indicated verbally that they could view the vocal cords. The vocal cords were recorded as seen if the participant indicated a Cormack and Lehane view of I or II when using the Macintosh blade. Time to successful intubation was defined as the time taken from insertion of the device into the ‘mouth’ of the manikin until the end of one successful lung inflation using an Ambu bag (Galemed®, I-Lan, Taiwan). After each intubation attempt the final ETT position was verified by the investigator.

Secondary endpoints recorded included the participants graded usability of each device rated on a scale from 1–3 (1, easy; 2, moderate; 3, difficult) and their device preference in each scenario. At the conclusion of the study participants were asked if they would use the iPhone adaptor for a fibreoptic intubation in their own practice if it were available and also to comment on the advantages and disadvantages of the iPhone in comparison to the other devices. All data was recorded by one of the two unblinded investigators.

Sample size was based on the duration of the successful tracheal intubation attempt. A literature review for trials using a fibreoptic intubation in a manikin and similar methodology demonstrated median intubation times ranging from 36 to 54 s. Similar patient trials have reported median times of 100 s with range of 54-195 s. Based on prior studies a difference of 20s in intubation time would be clinically important. To detect difference with power of 90%, for a significance level of 5%, we would require 21 subjects per group [8,9].

### Statistics

Statistical analysis was performed and graphs generated using SPSS 20 (IBM). The strength of the relationship between dependent variables (time to view the vocal cords and time to successful intubation) was assessed using the general linear regression model against independent factors such as intubation aid, operator experience, and intubation difficulty. Bonferroni’s *post hoc* analysis considered the difference between the four intubation aids. Data for subjective performances and preferred intubation aid were analyzed using Kruskal-Wallis one-way analysis of variance. The rate of intubation failure was analyzed by a Pearson Chi-square test. A P value < 0.05 was considered significant for all analyses [10,11].

## Results

**Figure 2:**
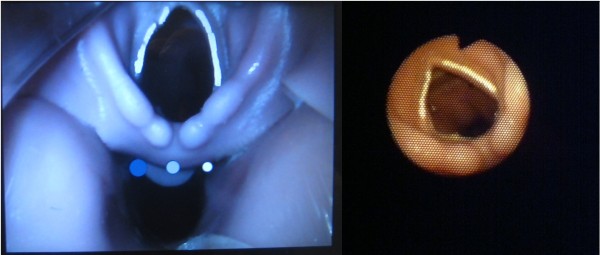
**Manikin vocal cords as visualized with the Glidescope (left) and iPMB (right).** The iPhone image was taken with camera zoom at 50% magnification.

A total of 63 anaesthesia providers, consisting of 3 groups; 21 consultant anaesthetists, anaesthetic registrars and anaesthetic nurses completed the study. Their levels of experience in clinical anaesthesia and with each intubation device used in this study are detailed in Table 1. The vocal cords as viewed with the Glidescope® and iPhone are shown in Figure 2.

**Table 1:** Characteristics and intubation experience of participants

	**Consultant**	**Registrar**	**Nurse**
Number of participants	21	21	21
Mean age (year)	46 (10)	33 (5)	45 (11)
Male: female ratio	15:6	11:10	6:15
Experience in anaesthesia (years)	17.6 (10)	3.4 (2)	8.9(8)
Estimated number of Macintosh intubations	All > 500	All > 50	3 (0–50)
Estimated number of Glidescope intubations	15 (0–200)	10 (0–30)	0 (0–3)
Estimated number of Fibreoptic intubations	20 (2–250)	0 (0–12)	0
Participants owning a smart phone (%)	71.4	95.2	76.2

Data reported as number, mean (SD) or median (range).

### Time to view the cords (TVC) and Time to successful intubation (TSI) versus device

**Figure 3:**
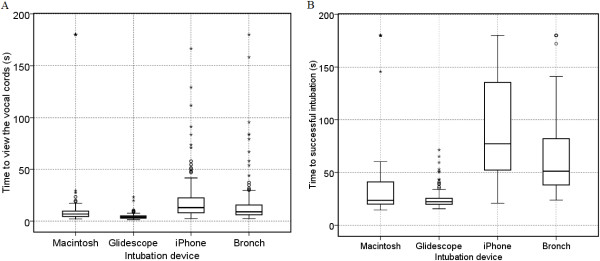
Boxplots demonstrating primary endpoints time to view the vocal cords (A) and time to successful intubation (B) for each intubation aid.

There was a significant difference (P < 0.001) between the four intubation devices and the primary end point dependent variables TVC and TSI (Figure 3A). The *post hoc* analysis (*Bonferroni*) for TVC demonstrated a significant difference between the Glidescope® and all other devices; Macintosh (P < 0.001), iPMFB (P < 0.001), and fibreoptic bronchoscope (P < 0.05). There was no statistically significant difference between the Macintosh and iPhone (P = 1.0) or Fibreoptic scope (P = 0.155). For TSI there was no significant difference between the Macintosh and fibreoptic bronchoscope (P =0.064). There were significant differences (P < 0.001) between all other intubate aids (Figure 3B).

### Time to view the cords and Time to successful intubation versus operator experience and simulated intubation difficulty

**Figure 4:**
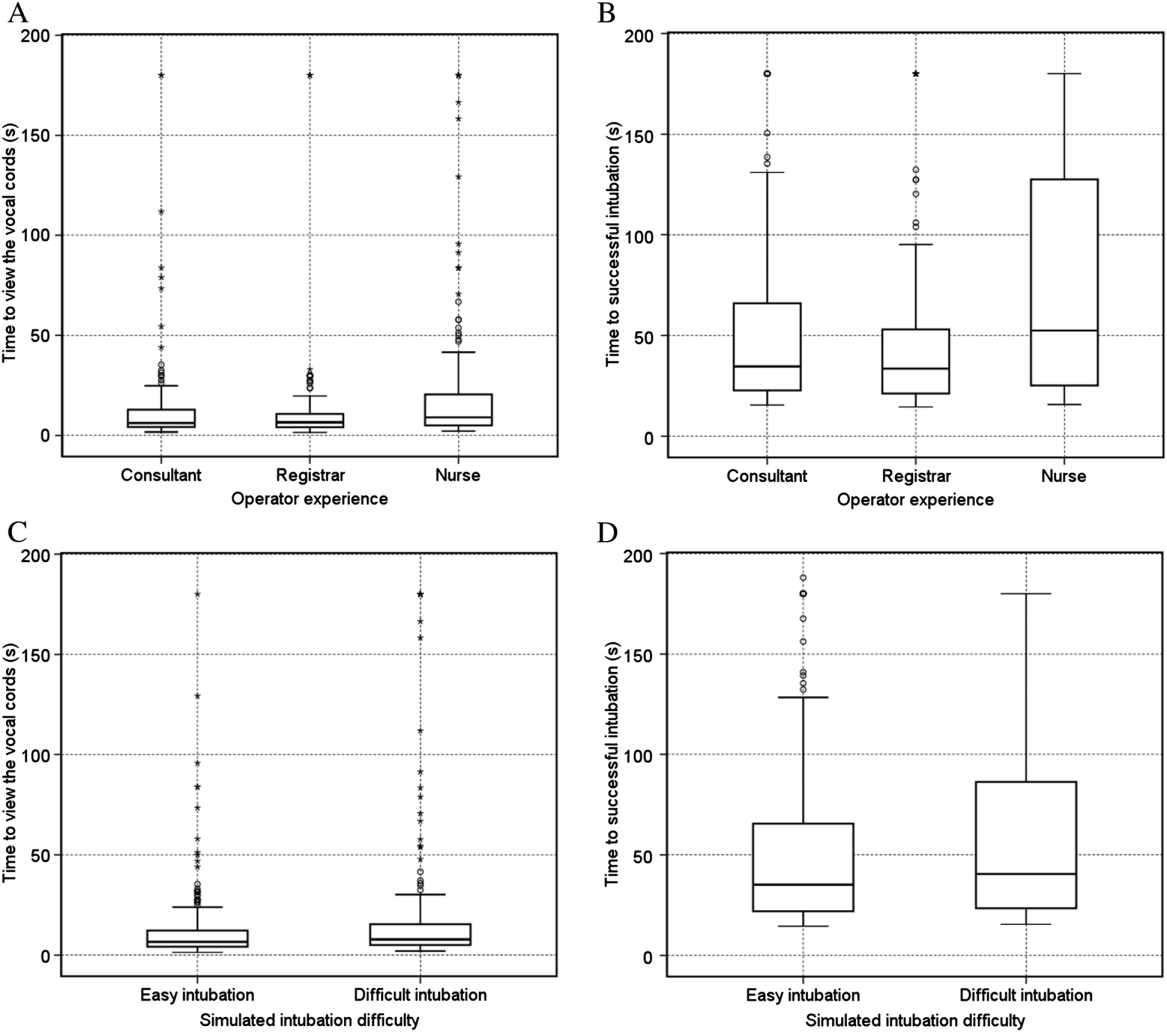
Boxplots demonstrating the effect of operator experience and intubation difficulty on time to view the vocal cords (A and C) and time to successful intubation (B and D).

There were significant differences (P < 0.001) for TVC and TSI versus experience of the operator (Figure 4A and B). The *post hoc* analysis showed that both consultant anaesthesist and registrars saw the vocal cords (P < 0.001) and intubated faster (P < 0.001) than the anaesthetic nurses. There was no significant difference between the consultants or registrars for TVC (P = 1.0) or TSI (P = 0.252). For simulated intubation difficulty there was a significant difference for TVC (P < 0.001) but no significant difference for TSI (P = 0.062) between the normal and difficult airway scenarios (Figure 4C and D).

### Failure to intubate

**Figure 5:**
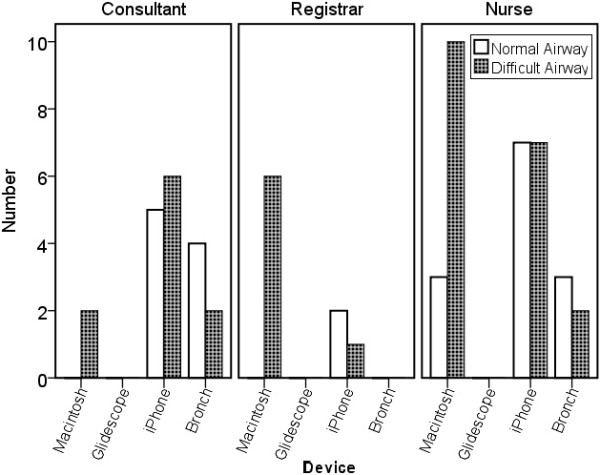
Graph representing the number of failed intubations per device for level of operator experience.

There were more failed intubations with the iPMFB, compared with the unmodified bronchoscope, in the consultant and nursing groups contrasting with no intubation failures with the Glidescope®. The registrars had fewer failed intubations with iPMFB compared with the unmodified bronchoscope. There was no significant difference between the groups in failure to intubate in the simulated normal airway (P = 0.324) or difficult airway (P = 0.118) (Figure 5).

### User rated device difficulty and preference

**Figure 6:**
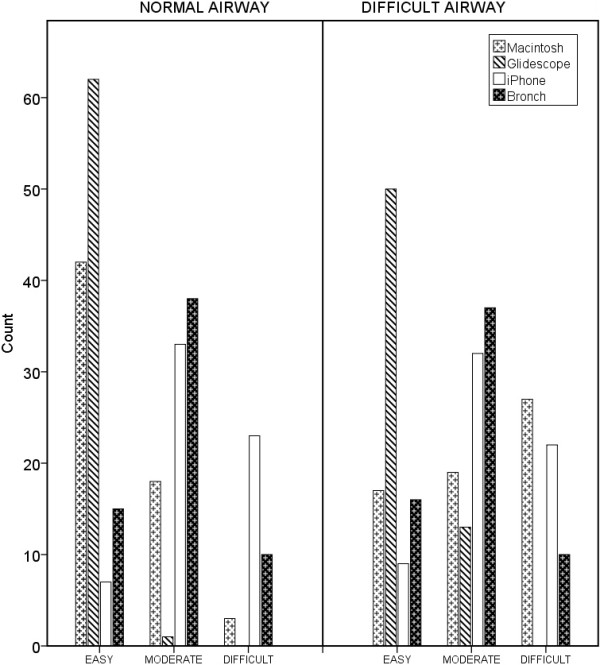
User rated device difficulty for normal and simulated difficult airway.

**Figure 7:**
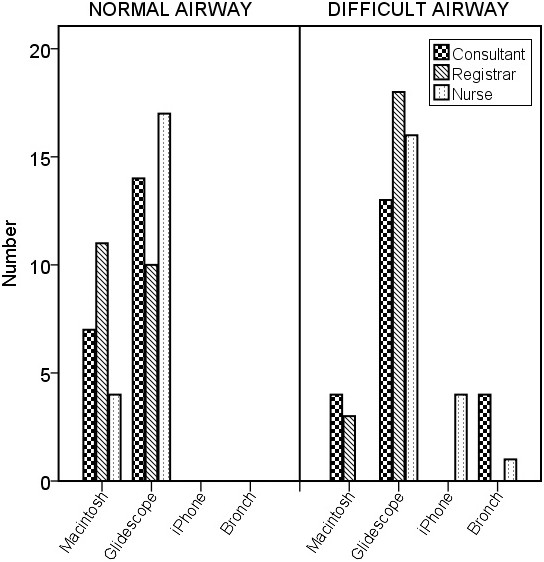
Device preference for normal and simulated difficult airway.

For the user rated device difficulty there was a significant difference (P < 0.001) with the Glidescope® being easier to use compared to the other devices (Figure 6). In the simulated normal airway the preference of the consultant anaesthetists and nurses was the Glidescope®. The registrars chose the Macintosh Laryngoscope over the Glidescope®. In the simulated difficulty airway most participants chose the Glidescope® as their preferred device (Figure 7). Similar proportions of consultants (14/21), registrars (15/21) and nurses (15/21) indicated that they would be prepared to use the iPMFB in their clinical practice.

## Discussion

Fibreoptic intubation is a clinically important technique requiring a high degree of manual dexterity and good psychomotor skills. Regular practice and training are required to maintain a high degree of competency [12]. This study has shown that the iPhone modification may be a useful adjunct to assist teaching fibreoptic intubation, but more experience is needed before it could be considered a clinically useful tool. TVC was similar between the Macintosh blade, iPMFB and unmodified bronchoscope. The Glidescope® demonstrated significantly less time to view the vocal cords compared to the other airway devices, which is consistent with previous studies [13,14].

TSI was significant longer with the iPMFB and less with the Glidescope® compared with the Macintosh blade or unmodified bronchoscope. We expected that the iPMFB might assist fibreoptic performance, through improved visualization of the upper airway anatomy using the larger, high resolution iPhone camera screen instead of the eye piece of the fibreoptic bronchoscope. This expectation was only partially supported by the results. Video assisted teaching has been shown to improve intubation success when teaching fibreoptic intubation [15]. Participants found that rapid identification of the vocal cords did not always translate into successful intubation.

The iPMFB was rated more difficult to use compared to the other airway devices and resulted in more failed intubations amongst consultant and nursing staff compared with the unmodified fibreoptic bronchoscope. Reasons for the poorer performance can be described in terms of ergonomics, optics and manikin related factors. The iPhone lens system attachment was heavy and added additional length to the fibreoptic bronchoscope, making it more difficult to use. Movement of the phone-lens-attachment apparatus resulted in distorted images requiring re-focusing and optimization of the camera position. The camera screen saver came on after prolonged intubation attempts, resulting in loss of image and was smaller than the Glidescope® screen. Glare reflecting into the camera lens from the fibreoptic light source made image acquisition difficult if the bronchoscope was against the plastic of the manikin. Despite these disadvantages, similar numbers of registrars, nurses and consultants reported that they would consider using the iPMFB for a fibreoptic intubation if the device was available. These opinions however may represent a novelty factor since the Glidescope® was rated as the easiest device to use in both airway scenarios and the preferred device by all participants in the simulated difficult airway scenario.

The use of manikins to simulate difficulty airway scenarios is widespread in anaesthesia; however, there is little evidence that these studies correlate with clinical performance [16,17]. In this study there was a significant difference between TVC in the normal versus the simulated difficult airway; but not in the TSI. This is unsurprising as participants rated the Macintosh blade more difficult to use when the hard collar was applied. Hard collars have been used in previous manikin studies to simulate a difficult airway scenario [18]. The Glidescope® and flexible fibreoptic scope have previously been shown to have similar times required for tracheal intubation in a study of patients with anticipated difficult airways [19].

When considering previous experience level upon performance, there was no significant difference between anaesthetic consultants and registrars in terms of the primary outcomes. The anaesthetic nurses were able to intubate the manikin with little training or experience using the Glidescope®, demonstrated by the lack of failed intubations in both airway scenarios. The registrars had fewer failed intubations using the iPMFB and unmodified bronchoscope compared to the consultants and nurses. This may be explained by the regular practice required to maintain a high degree of dexterity and fibreoptic competence. Although the consultants had performed more fibreoptic intubations overall the trial facility lacked a surgical service requiring regular fibreoptic intubations. Many of the participating training registrars had rotated through a nearby tertiary hospital and may have had more recent training and practice compared to their senior colleagues. Some registrars had had no previous fibreoptic experience but appeared to be more comfortable using bronchoscope with the iPhone attachment compared with the senior clinicians used to viewing through the eye piece. Consultant anaesthetists performed better than both registrars and nurses with the Macintosh blade, supporting the construct validity of our model.

The major limitation of this study was its conduct utilizing a manikin simulator in a controlled environment and not patients in a clinical scenario. The manikin setup was constant between participants which is rare clinically. There was also potential for bias in the methodology and conduct of the trial; participants and assessors were not blinded to the device used and the primary endpoint was dependent upon the participant verbalizing when the vocal cords were visualized. The results may have been different if a smaller size or brand of ETT was used or if it were conducted in a facility where fibreoptic intubations are routinely performed. Importantly, participants were aware they were being timed which can impact the validity of the results by the Hawthorne and Rosenthal effects. The Hawthorne effect, the awareness of being under observation, can influence the behaviour of the participant, potentially altering their performance with each device or the investigators instructions to the participant. The Rosenthal effect occurs when the outcome of the study is skewed when the participant responds based on an awareness of the experimenters expectations [20]. More clinically useful information may have been obtained from a comparison of the iPMB, unmodified bronchoscope attached to a video screen and direct viewing through the bronchoscope eye piece or iPhone image streamed to an iPad to mimic a video stack.

Despite the limitations of the iPMFB in this study mobile phones may have a role in teaching fibreoptic intubation technique. Smart phones are widely available, relatively inexpensive, and are more portable than a video stack. The study device was easy to set up and offered the ability to manipulate the image using the camera functions in real time. The lens system can be used to connect any smart phone to a bronchoscope by removing the lens adaptor and incorporating this into a hard back plastic phone cover for the mobile. The Wi-Fi and 3G capability can enable streaming of images to a Wi-Fi compatible monitor, smart device or tablet, across the internet or to a website.

## Conclusion

In this manikin simulation the iPhone modified bronchoscope resulted in similar times to view the vocal cords but significantly longer times to successful intubation compared with the Macintosh blade and unmodified bronchoscope The combination of smart phone technology and fibreoptics may provide an alternate and relatively inexpensive method of teaching this fibreoptic skills however further research is required to assess its usefulness as an intubation aid in a clinical scenario.

## Competing interests

The authors had no competing interests in the performance of this study.

## Authors’ contributions

AL designed the study, tested the subjects, analyzed the data and composed the manuscript. GM helped with study design, ethics approval, data interpretation, equipment, manuscript review, and subject testing. Both authors read and approved the final manuscript.

## Pre-publication history

The pre-publication history for this paper can be accessed here:

http://www.biomedcentral.com/1471-2253/14/10/prepub
